# The 80^th^ Threonine Residue of Histone H3 Is Important for Maintaining HM Silencing in *Saccharomyces cerevisiae*

**DOI:** 10.4014/jmb.2310.10031

**Published:** 2023-11-15

**Authors:** Soojin Yeom, Junsoo Oh, Donghyun Kim, Jung-Shin Lee

**Affiliations:** 1 Department of Molecular Bioscience, College of Biomedical Science, Kangwon National University, Chuncheon 24341, Republic of Korea; 2 Institute of Life Sciences, Kangwon National University, Chuncheon 24341, Republic of Korea

**Keywords:** *Saccharomyces cerevisiae*, gene silencing, histone threonine residue, homothallic mating loci, telomere

## Abstract

Gene expression in eukaryotic cells is intricately regulated by chromatin structure and various factors, including histone proteins. In *Saccharomyces cerevisiae*, transcriptionally silenced regions, such as telomeres and homothallic mating (HM) loci, are essential for genome stability and proper cellular function. We firstly observed the defective HM silencing in alanine substitution mutant of 80^th^ threonine residue of histone H3 (H3T80A). To identify which properties in the H3T80 residue are important for the HM silencing, we created several substitution mutants of H3T80 residue by considering the changed states of charge, polarity, and structural similarity. This study reveals that the structural similarity of the 80^th^ position of H3 to the threonine residue, not the polarity and charges, is the most important thing for the transcriptional silencing in the HM loci.

## Introduction

In eukaryotes, gene expression and genome stability are regulated in the context of the chromatin structure, which is classified into the euchromatin or heterochromatin depending on the levels of the chromatin condensation [1-4]. The highly condensed nature of the heterochromatin regions prevents the underlying DNA sequences from the accesses of the factors for recombination and transcription, thereby characteristically leading the transcriptional silencing [5, 6]. In *Saccharomyces cerevisiae*, typical components of the heterochromatin including H3K9 methylation and HP1 (Heterochromatin Protein 1) homologs don’t exist. Instead, transcriptional silencing determined by the spreading of SIR (Silent Information Regulator) complex consisting of the Sir2, Sir3, and Sir4, is shown in some regions including telomeres, homothallic mating (HM) loci, and rDNA regions [3, 7-11].

The transcriptional silencing in these regions is affected by both the histone residue itself and residue-specific histone modifications. For examples, H3K4 methylation, H3K79 methylation, and H2B ubiquitination are suggested to be important for the transcriptional silencing of telomeres [12-17]. While alanine substitution (H3K79A) of the H3K79 residue compromised the HM silencing, both mutants substituting the lysine residue of histone H3 with same positively charged arginine (H3K79R) and deleting the cognate methyltransferase (*Δdot1*) maintain HM silencing well [17]. These results suggest that in case of the HM silencing, not the H3K79 methylation, but the property of the 79^th^ residue itself of histone H3, including the positive charge, is important.

*S. cerevisiae* is a good model organism to identify the histone residues and modifications relevant to the transcriptional silencing. A previous study made the scanning histone mutagenesis with alanine (SHIMA) library, each strain of which substitutes each residue of histone H2A, H2B, H3, and H4, with alanine [20]. Also, in budding yeast, whether a yeast strain has defects in the HM silencing is easily identified through the treatment of α-factor (alpha-factor) to the a-type mutants [17]. The mating type of a haploid yeast cell is determined by the mating type gene, either “a” or “α (alpha)”, inserted in the *MAT* locus in chromosome III. The *MAT* locus is surrounded by two genes, *HMLalpha* and *HMRa*, encoding mating type genes of “α (alpha)” and “a”, respectively. Since both are maintained in the transcriptionally silenced state, the regions are called as the homothallic mating (HM) loci. Haploid *MATa* cells express a-type specific factors including α-factor receptor *STE2*, which lead to the cell-cycle arrest in the G1 phase [17]. In contrast, when HM silencing is disrupted, genes at the cryptic mating loci are expressed, which represses the expression of a-specific genes including *STE2* [17]. As the result, the mutants grow normally even in the α-factor-containing media [17-19].

In this study, each of the histone point mutant library, which substitutes each residue of histone H2A, H2B, H3, and H4, with alanine, was spread on the YPD media with disks containing the different concentration of α-factor. As the result, we isolated the H3T80A mutant showing the phenotype of HM silencing defects. To further identify which properties of the T80 residue are important for the HM silencing, we constructed several strains substituting the T80 residue with negative charged (D, E), polar uncharged (S), hydrophobic (V, I, F, Y), and cysteine (C) residues. The amino acids with structural similarity to the threonine, including C, I, V, and S, could substitute the threonine for the Sir2-dependent regulation of HM silencing. In contrast, either the negatively charged amino acids (D, E) or aromatic amino acids (F, Y) cannot replace the T80 residue for the HM silencing. In line with the well-documented association of H3K79 residue to silencing, we confirmed the mutants capable of substituting the K79 residue in HM silencing [17]. Taken together, we suggest that the structural integrity of the H3T80, neighboring the K79 residue and positioned in the L1 loop of histone H3, is crucial for the maintenance of HM silencing.

## Materials and Methods

### Histone Point Mutant Mutagenesis

Site-directed mutagenesis was performed on the pBL267 plasmid using QuickChange Site-Directed Mutagenesis Kit (Agilent Technologies, USA, Cat# 200519). The primers used for the construction of histone point mutants are listed in Table S2, and the genotypes of the resulting histone point mutants are listed in Table S1.

### α-Factor Screening and Serial Dilution Assay

After plating 100 μL of cells diluted into 10^6^ cells/ml on the YPD plates, three sterilized paper disks were placed on three points on the plate. 5 μl α-factor of three different concentrations (1 μg/μl, 0.1 μg/μl and 0.01 μg/μl, respectively) was spotted onto the paper disks and the plates were incubated at 30°C for 3 days. H4K16A mutant strain was used as a negative control, and wild-type (WT) was used as a positive control. To confirm more sensitive result, each mutant diluted into 10^6^-10^3^ cells/ml and spotted onto the non-treated YPD and 0.05 μg/μl α-factor treated YPD.

### Chromatin Immunoprecipitation (ChIP)

Each mutant cells were grown to OD_600_ = 1.0 in 100 ml YPD. Cells were lysed by beadbeating in the lysis buffer (50 mM HEPES pH 8.0, 140 mM NaCl, 1 mM EDTA, 1% Triton X-100, 0.1% sodium deoxycholate) at 4°C and chromatin was sheared by sonication. 200 ul chromatin extract and 200 ul lysis buffer were incubated with anti-sir2 antibody (santa cruz biotechnology, 200 ug/ml, Cat# SC-25753) and immunoprecipitated by A/G agarose beads. Both input and immunoprecipitated chromatin were reverse-crosslinked and the DNA was extracted by the PCI (Phenol:chloroform:isoamyl alcohol (25:24:1)) extraction. Immunoprecipitated DNA was dissolved in TE (Tris-EDTA) buffer.

### Quantitative PCR (q-PCR)

The qPCR primers targeting the *HML1* and *HML4* loci are listed in the Table S2. Quantitative PCR (q-PCR) was performed using the TOPreal qPCR 2x premix (SYBR green with low ROX, Enzynomics Cat# RT500M). qPCR experiments were performed as the manufacturer’s protocol.

## Results

### Substitution of H3T80 with Negatively Charged Amino Acids Cannot Replace the Threonine for HM Silencing

**Fig. 1:**
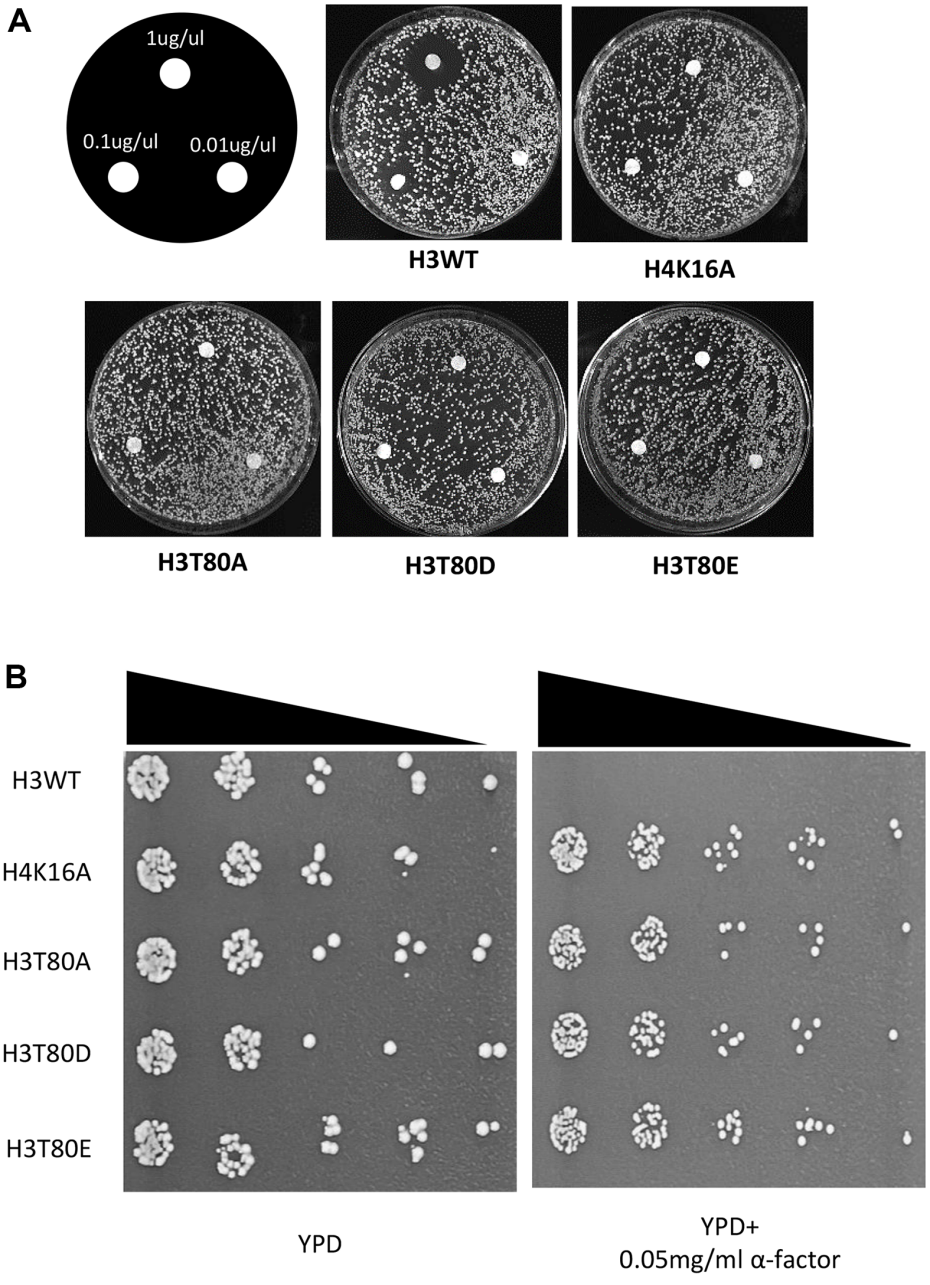
Substitution of H3T80 with negatively charged amino acids cannot replace the threonine for HM silencing. (**A**) Substitution of 80^th^ threonine with alanine or two negatively charged amino acids (H3T80D and H3T80E) leads to loss of HM silencing. The clear zone, formed in the vicinity of the α-factor containing disks, disappeared in H3T80A, H3T80D, and H3T80E strains, indicating loss of HM silencing. (**B**) HM silencing defects resulting from substitutions of the 80^th^ threonine of histone H3 with alanine, aspartic acid, or glutamic acid were reproducibly observed in a serial dilution assay.

A previous study made the scanning histone mutagenesis with alanine (SHIMA) library, each strain of which substitutes each residue of histone H2A, H2B, H3, and H4, with alanine [20]. In this study, to identify the histone residues relevant to the HM silencing, we performed the α-factor screening with each strain of the SHIMA library and isolated the H3T80A mutant (Data not shown). To validate the results, we plated the H3T80A mutant in the YPD plate with disks containing different concentrations of α-factor. Wildtype (H3WT) strain shows the round clear zone around the α-factor disks resulting from the maintenance of HM silencing and arrested cell cycle (Fig. 1A). In contrast, the clear zone disappeared in the H3T80A mutant similar to the H4K16A control strain, suggesting that the strain is insensitive to the α-factor and the HM silencing is defective (Fig. 1A). H4K16A mutant was used as the control strain since the strain is well-known to have defects in HM silencing [21, 22]. Also, the serial dilution assay reconfirmed that H3T80A mutant is insensitive to the addition of α-factor (Fig. 1B).

To identify whether substitution of the 80^th^ residue of H3 could restore the HM silencing defects in the H3T80A mutant, we firstly replaced the 80^th^ residue of H3 with the two negatively charged amino acids, aspartic acid (D), and glutamic acid (E). The two assays show that both mutants (H3T80D and H3T80E) didn’t maintain the HM silencing well (Fig. 1A and 1B). So, these results suggest that the presence of negative charge in the 80^th^ residue of histone H3 inhibits the maintenance of the HM silencing in *S. cerevisiae*.

### The Structural Similarity of the 80^th^ Residue in Histone H3 to Threonine is Important for Maintaining HM Silencing

**Fig. 2:**
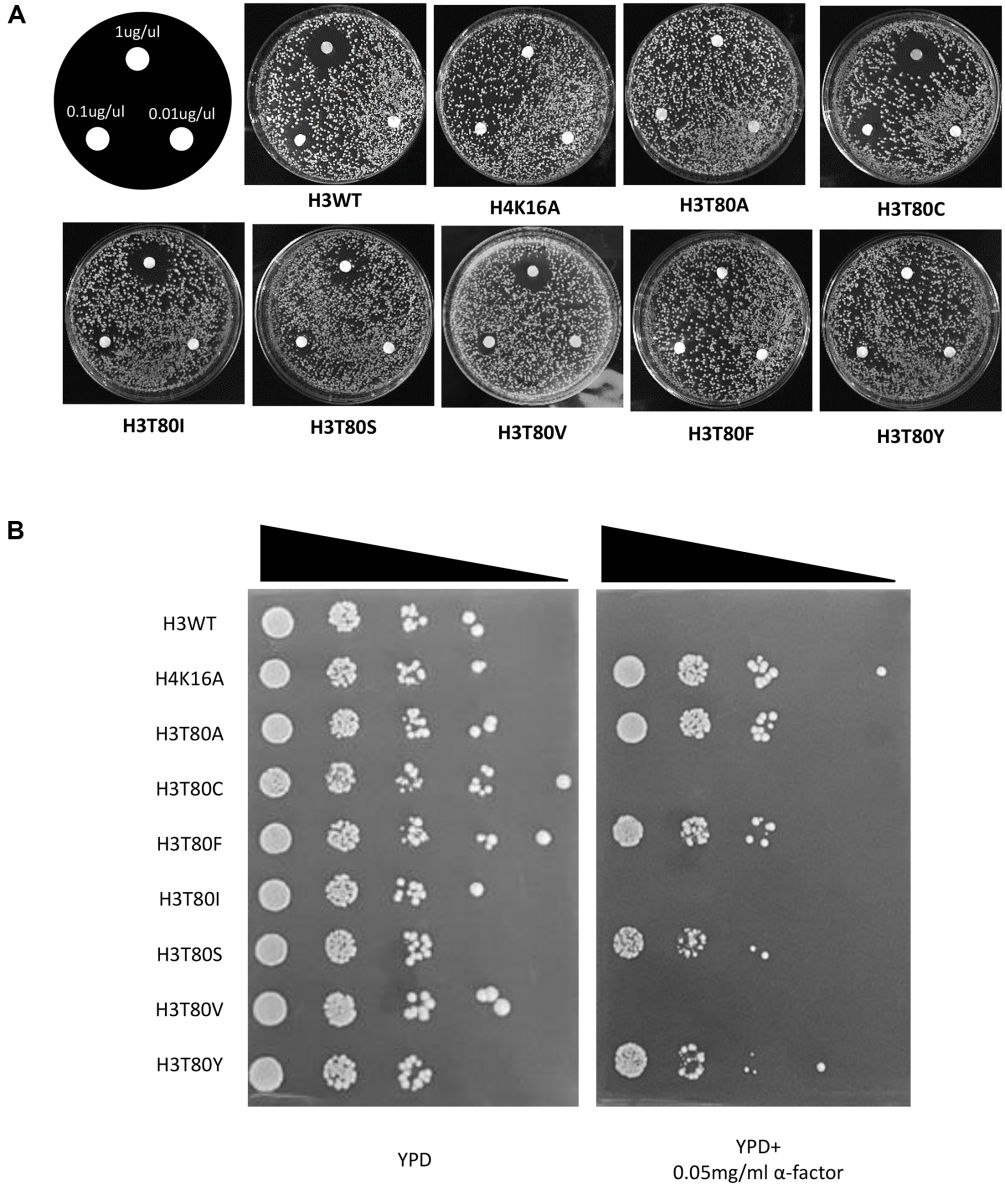
The structural similarity of the 80^th^ residue in histone H3 to threonine is important for maintaining HM silencing. (**A**) Substitution of 80^th^ threonine residue of histone H3 with structurally similar residues, including C, I, S, and V replaced the role of 80^th^ threonine of histone H3 in the HM silencing. As described in Fig. 1A, a disk assay was performed with substitution mutants of H3T80, including H3T80A, H3T80C, H3T80I, H3T80S, H3T80F, and H3T80Y. (**B**) A serial dilution assay was performed as described in Figure1B. This assay reconfirmed the results in Figure 2A except for the H3T80S strain which shows the growth in the media containing low concentration of α-factor (0.05 μg/μl).

To identify if the hydrophobicity in the substituted alanine leads the loss of HM silencing in the H3T80A strain, we created the substitution mutants of H3T80 with some hydrophobic amino acids, isoleucine (I), valine (V), phenylalanine (F), and tyrosine (Y). While substitution with isoleucine (I) and valine (V) successfully replaced the 80^th^ threonine residue for the HM silencing, the substitution with aromatic ring-containing amino acids, including phenylalanine (H3T80F) and tyrosine (H3T80Y), disturbed the HM silencing (Fig. 2A and 2B). The strain substituting the 80^th^ threonine with the same polar amino acid serine (H3T80S) showed opposite results in both assays (Fig. 2A and 2B). The H3T80S strain grew well in the serial dilution assay which means that the strain is insensitive to the lower α-factor concentration (0.05 mg/ul). However, the strain didn’t grow around the α-factor-containing disk (1 ug/ul) means that the strain shows sensitivity to much higher concentration of α-factor (1 ug/ul). So, the extent of HM silencing in the H3T80S strain is intermediate between the wildtype strain and HM silencing defective mutants. Interestingly, when the hydroxyl group of the serine is changed with the thiol group (Cysteine), the H3T80C mutant successfully restored the role of the 80^th^ threonine in HM silencing (Fig. 2A and 2B). The features shared in common among the substitution mutants that replaced the role of the 80^th^ threonine in the HM silencing, including C, I, S, and V, are the structural similarity to the threonine residue, not the polarity or charge.

More specifically, we think that either the hydroxyl group (-OH) or γ-carbon (gamma carbon) should not be lost from the β-carbon (beta carbon) of the 80^th^ threonine residue of histone H3. While loss of both compromised the HM silencing (H3T80A), loss of only γ-carbon (H3T80S) or replacement of the hydroxyl group (-OH) with a carbon (H3T80V), and addition of a carbon to the valine (H3T80I) successfully replaced the threonine for the HM silencing maintenance. However, addition of an aromatic ring (H3T80F or H3T80Y) or a negative charge (H3T80D or H3T80E) compromised the HM silencing. Taken together, the loss of both γ-carbon and hydroxyl group, addition of an aromatic ring or negative charge to the 80^th^ residue of histone H3 must be prevented to maintain the HM silencing.

### Structurally Similar Residues in the 80^th^ Amino Acid of Histone H3 Enable enough Chromatin Occupancy of the Sir Complex

**Fig. 3:**
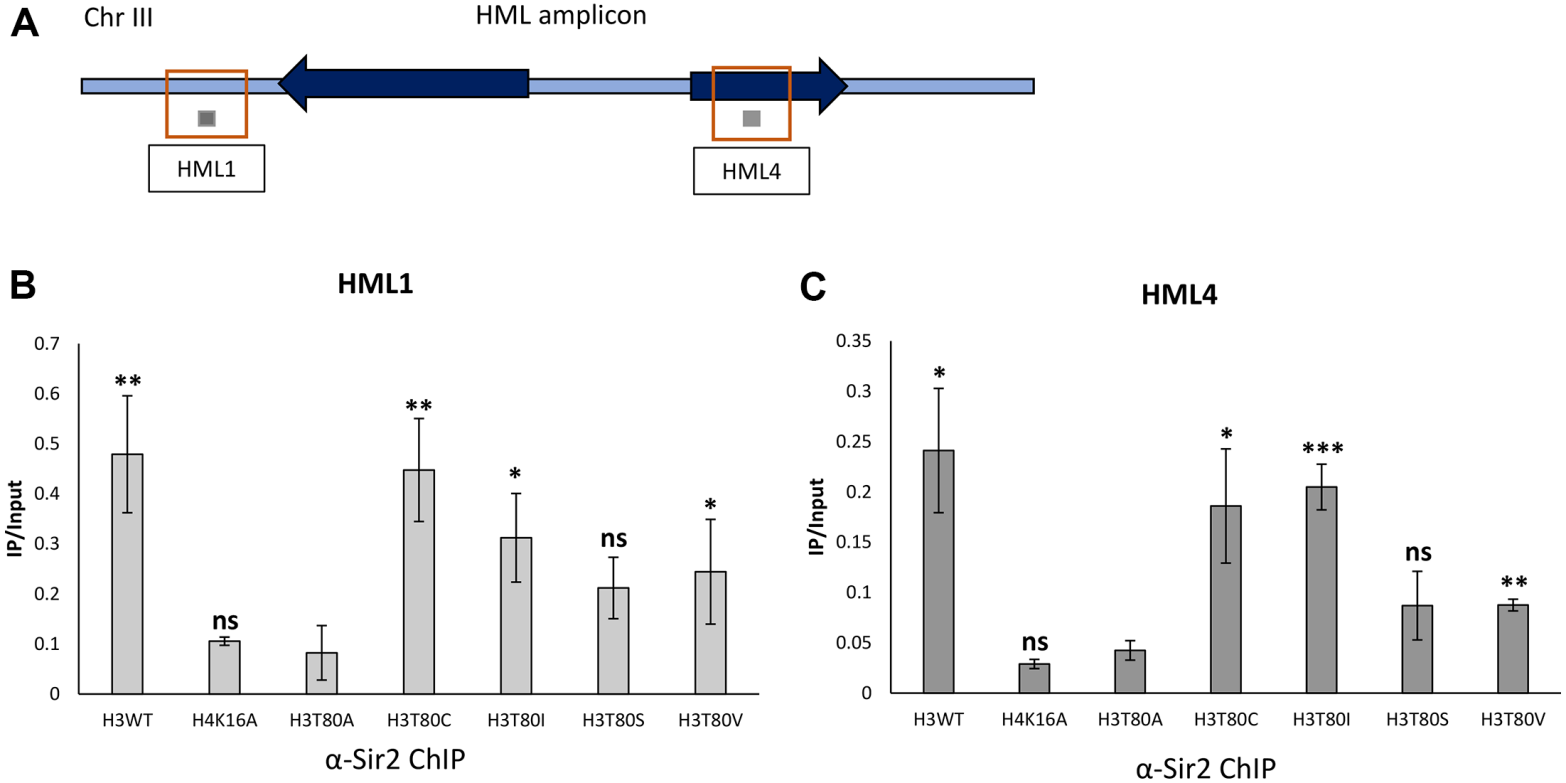
Structurally similar residues in the 80^th^ amino acid of histone H3 to the threonine enable enough chromatin occupancy of the Sir complex. (**A**) Schematic representation of the chromatin immunoprecipitation (ChIP) q-PCR primers targeting *HML1* and *HML4* of the *HML* region localized in chromosome III. *HML1* and *HML4* are represented as the lined boxes, and the amplicons of *HML1* and *HML4* are depicted as the gray boxes. (**B** and **C**) ChIP analyses with the antibody against the Sir2 were carried out with mutant strains in which the 80^th^ threonine of histone H3 were substituted with alanine, cysteine, isoleucine, serine or valine. qPCR experiments with primers targeting the (**B**) *HML1* and (**C**) *HML4* were performed and the IP values were normalized with the input values. The bar graph shows the average normalized value of three independent experiments and the calculated *p*-values designate the significance of the differential Sir2 binding compared to the H3T80A strain (*p < 0.05, **p < 0.01, ***p < 0.001).

To identify whether the structurally similar residues in the 80^th^ amino acid of histone H3 enabled the enough occupancy of Sir complex on the *HML* loci to maintain the HM silencing, we performed chromatin immunoprecipitation (ChIP) using an antibody against Sir2, a subunit of the SIR complex. As expected, the occupancy of Sir2 on the *HML* loci severely reduced in the two HM silencing defective strains, H4K16A and H3T80A (Fig. 3A-3C). On contrary, the occupancy of Sir2 on the *HML* loci increased to some extents in the strains that maintain the HM silencing, including H3T80C, H3T80I, and H3T80V, albeit the restored occupancy of Sir2 is not enough to compare with the wildtype strain (Fig. 3B and 3C). Also, the H3T80S strain which is only sensitive to the higher concentration of α-factor has relatively lower occupancy of Sir2 than H3T80C, H3T80I, and H3T80V strains. These results suggest that the structure similarity of the 80^th^ residue of the histone H3 to the threonine determines the occupancy of Sir complex on the HM loci for the maintenance of HM silencing.

### Integrity of the Two Consecutive Amino Acids, H3K79-H3T80, is Crucial for the HM Silencing and Tri-Methylation of H3K79

In *S. cerevisiae*, all H3K79 methylations including mono-, di-, and tri-methylation are modified by Dot1, and the Sir3, the core component of yeast Sir complex, is not bound to the H3K79 methylated nucleosome [23-26]. In other words, the Dot1 competes with Sir3 to bind to the nucleosome. In this study, occupancy of Sir2 was reduced even in the strains maintaining the sensitivity to the α-factor including substitution mutants of T80 of H3 with C, I, S, or V, as well as the mutants losing the HM silencing (H3T80F, Y, D, or E) (Fig. 1-3).

To identify whether the reduction of the Sir2 occupancy in these strains result from the increase in the methylation of H3K79, the neighboring residue of H3T80, we confirmed the H3K79 methylation levels of these H3T80 substitution mutants by western blot (Fig. S1). The H3K79me3 levels in most mutants significantly reduced except for the two strains (H3T80I and H3T80V). In addition, H3K79me2 level was also decreased in case of the H3T80F strain. Taken together, the cause of the reduced Sir complex occupancy in the H3T80 mutants is at least not from the increased H3K79 methylation levels. Also, considering both isoleucine (I) and valine (V) have the most similar structure to the threonine (T), structural similarity of the 80^th^ amino acid of histone H3 to the threonine (T) looks crucial for both HM silencing and H3K79me3.

**Fig. 4:**
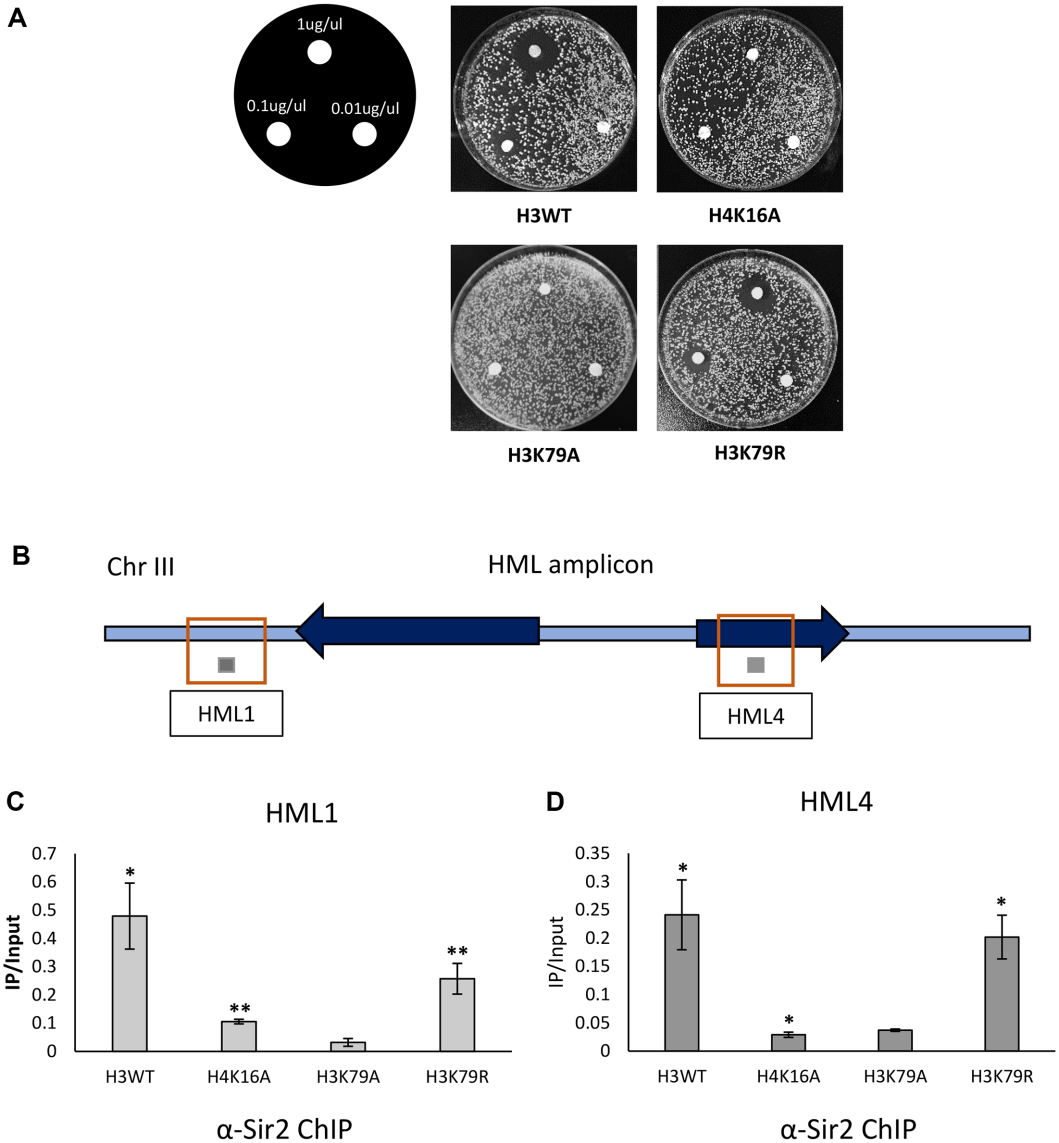
Integrity of the 79^th^ lysine residue of histone H3 is crucial for the SIR complex-dependent HM silencing. (**A**) Substitution of the 79^th^ lysine residue of histone H3 with arginine (H3K79R) replaced the role of the 79^th^ lysine residue of histone H3 in the HM silencing, but with alanine (H3K79A) did not. As described in Fig. 1A, a disk assay was performed with substitution mutants of H3K79, including H3K79A and H3K79R. (**B**) Schematic representation of the chromatin immunoprecipitation (ChIP) q-PCR primers targeting *HML1* and *HML4* of the *HML* region localized in chromosome III. *HML1* and *HML4* are represented as the lined boxes, and the amplicons of *HML1* and *HML4* are depicted as the gray boxes. (**C** and **D**) ChIP analyses with the antibody against the Sir2 were carried out with mutant strains in which the 79^th^ lysine of histone H3 were substituted with alanine (H3K79A) or arginine (H3K79R). qPCR experiments with primers targeting the (**C**) *HML1* and (**D**) *HML4* were performed and the IP values were normalized with the input values. The bar graph shows the average normalized value of three independent experiments and the calculated *p*-values designate the significance of the differential Sir2 binding compared to the H3K79A strain (*p < 0.05, **p < 0.01, ***p < 0.001).

A previous study suggested that the H3K79 residue itself, not the H3K79 methylation, is important for the HM silencing, based on the results that both the *Δdot1* and *H3K79R* strain maintain HM silencing well, but the H3K79A strain lost its HM silencing [17]. So, to identify and confirm whether integrity of H3K79 residue is also crucial for the HM silencing, we performed the disk assay with both the H3K79A and H3K79R strains. Coinciding with the previous result, H3K79R strain maintains HM silencing, but H3K79A strain doesn’t (Fig. 4A). Sir2 ChIP-qPCR experiments show that while the H3K79A mutant lost significant occupancy of Sir2 on the *HML* loci, significant occupancy of Sir2 on the *HML* loci was detected in the H3K79R strain (Fig. 4B). Taken together, we suggest that the structural integrity of the two consecutive amino acids, H3K79 and H3T80, is crucial for the transcriptional silencing of the HM loci by recruitment of Sir complex.

## Discussion

The importance of L1 loop of histone H3 encompassing the H3K79 and H3T80 residues for the yeast transcriptional silencing was already suggested through a previous study [27]. Also, another study identified that the H3T80A mutant shows the silencing defects in both telomeres and HM loci [28]. Nevertheless, by substituting the H3T80 residue with amino acids with more various properties, including different polarity, charge, and shapes, we appreciated the importance of the structural integrity of the H3T80 residue for the heterochromatin formation and transcriptional silencing.

This study firstly suggests the role of potential H3T80 phosphorylation (H3T80ph) for the inhibition of silencing factor enrichments in the silent loci in *S. cerevisiae*. The phosphorylation mimetic H3T80D and H3T80E mutants showed the reduction of both H3K79me3 level and the HM silencing defects which result from the reduced occupancy of the silencing factor, SIR complex (Figs. 1 and S1). A previous study revealed the existence of the H3T80ph in metazoan including mammals and drosophila, although they could not detect the H3T80ph in both *S. cerevisiae* and *S. pombe* [13]. Nevertheless, based on the sequence conservation encompassing the L1 loop of histone H3 including the H3K79 and H3T80 residue in yeast and mammals, it is likely that the H3T80ph in the higher eukaryotes also regulate the heterochromatin structure formation. In line with this, interactions between neighboring nucleosomes and chromatin compaction during the mitosis were suggested to be promoted by H3T80 phosphorylation in the metazoan cells [13]. It looks counterintuitive that H3T80 phosphorylation inhibits the silent factor occupancy and silent chromatin formation in yeast, but promotes the chromatin compaction in higher eukaryotes. However, H3S10 phosphorylation, another example of methylatable lysine - phosphorylatable residue coupling, excluded the representative silencing factor HP1 (Heterochromatin Protein 1) from both the neighboring H3K9 methylation and chromosomes during the metaphase [29]. So, further studies are required to identify whether occupancy of HP1 to the heterochromatin is also inhibited by H3T80ph and the changed status of HP1 binding by H3T80ph is connected to the chromatin compaction.

H3K79, the neighboring residue of H3T80, is usually methylated by Dot1 and DOT1L in *S. cerevisiae* and mammals, respectively [26]. In *S. cerevisiae*, either deletion or overexpression of Dot1, or H3K79A mutation compromises the telomeric silencing by disrupting the sir complex localization [15]. In particular, methylation of H3K79 prevents the binding of Sir3 to the histone both in vitro and in vivo [30, 31]. In contrast, although the H3K79A mutant shows the HM silencing defects, either H3K79R mutant or ablation of H3K79 methylation did not compromise the HM silencing [17] (Fig. 4). Taken together with these results and the data in this study, the integrity of the side chain in the consecutive amino acids, H3K79-H3T80, is crucial for the occupancy of the Sir complex, thereby maintaining the transcriptional silencing in the HM loci.

We suggest that the minute structural changes in the two consecutive amino acids H3K79-H3T80 trigger the vast structural changes in the chromatin structure. Specifically, our data showed that the loss of local transcriptional silencing accompanied with the loss of silencing factors, Sir2. Actually, mutations of the mammalian H3K79 methyltransferase DOT1L are recurrently found in several types of solid cancers, such as melanoma, colorectal cancer and ovarian cancer [32]. So, the structures of H3K79 and H3T80 residues must be protected from mutations to prevent the various pathologies resulting from the vast chromosomal rearrangements.

There are also some limitations in this study. Although we revealed that the similarity of the 80^th^ residue of histone H3 to the threonine is crucial for the HM silencing, we don’t know exactly why the Sir complex occupancy, H3K79 methylation, and the HM silencing were decreased by the minute structural change of the 80^th^ threonine of histone H3. More detailed in vitro structural studies using the recombinant nucleosomes containing the H3T80 mutation and the recombinant Sir complex would give the answers to this question.

## Supplemental Materials

Supplementary data for this paper are available on-line only at http://jmb.or.kr.
