# Supplementary material for: The 80th Threonine Residue of Histone H3 Is Important for Maintaining HM Silencing in Saccharomyces cerevisiae
Source: J Microbiol Biotechnol. 2023 Nov 15;34(1):39–46. doi: 10.4014/jmb.2310.10031 (PMC10840469; doi:10.4014/jmb.2310.10031)
Supplement: Supplementary file 1 [file jmb-34-1-39-supple.pdf]

J. Microbiol. Biotechnol.
https://doi.org/10.4014/jmb.2310.10031

Supplementary Figure and Tables

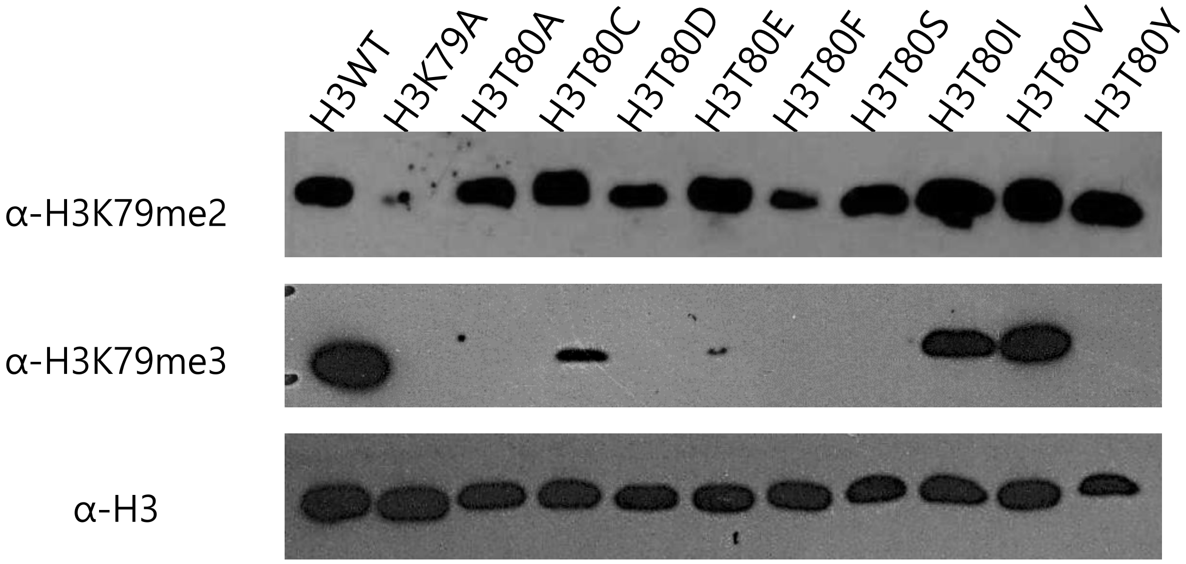

Fig. S1. Maintenance of H3T80 structure is correlated with H3K79me3

Table S1. Yeast strains used in this study.

Name	Description
H3WT	MATa leu2Δ1 his3Δ200 ura3-52 trp1Δ63 lys2-1286 (hht1-hhf1)ΔLEU2 (hht2-hhf2)Δ::HIS3 Ty912Δ35-lacZ::his4 pHHT1-HHF1::TRP1
H4K16A	MATa leu2Δ1 his3Δ200 ura3-52 trp1Δ63 lys2-1286 (hht1-hhf1)ΔLEU2 (hht2-hhf2)Δ::HIS3 Ty912Δ35-lacZ::his4 pHHT1-HHF1R16A::TRP1
H3K79A	MATa leu2Δ1 his3Δ200 ura3-52 trp1Δ63 lys2-1286 (hht1-hhf1)ΔLEU2 (hht2-hhf2)Δ::HIS3 Ty912Δ35-lacZ::his4 pHHT1K79A-HHF1::TRP1
H3K79R	MATa leu2Δ1 his3Δ200 ura3-52 trp1Δ63 lys2-1286 (hht1-hhf1)ΔLEU2 (hht2-hhf2)Δ::HIS3 Ty912Δ35-lacZ::his4 pHHT1K79R-HHF1::TRP1
H3T80A	MATa leu2Δ1 his3Δ200 ura3-52 trp1Δ63 lys2-1286 (hht1-hhf1)ΔLEU2 (hht2-hhf2)Δ::HIS3 Ty912Δ35-lacZ::his4 pHHT1T80A-HHF1::TRP1
H3T80C	MATa leu2Δ1 his3Δ200 ura3-52 trp1Δ63 lys2-1286 (hht1-hhf1)ΔLEU2 (hht2-hhf2)Δ::HIS3 Ty912Δ35-lacZ::his4 pHHT1T80C-HHF1::TRP1
H3T80D	MATa leu2Δ1 his3Δ200 ura3-52 trp1Δ63 lys2-1286 (hht1-hhf1)ΔLEU2 (hht2-hhf2)Δ::HIS3 Ty912Δ35-lacZ::his4 pHHT1T80D-HHF1::TRP1
H3T80E	MATa leu2Δ1 his3Δ200 ura3-52 trp1Δ63 lys2-1286 (hht1-hhf1)ΔLEU2 (hht2-hhf2)Δ::HIS3 Ty912Δ35-lacZ::his4 pHHT1T80E-HHF1::TRP1
H3T80F	MATa leu2Δ1 his3Δ200 ura3-52 trp1Δ63 lys2-1286 (hht1-hhf1)ΔLEU2 (hht2-hhf2)Δ::HIS3 Ty912Δ35-lacZ::his4 pHHT1T80F-HHF1::TRP1
H3T80I	MATa leu2Δ1 his3Δ200 ura3-52 trp1Δ63 lys2-1286 (hht1-hhf1)ΔLEU2 (hht2-hhf2)Δ::HIS3 Ty912Δ35-lacZ::his4 pHHT1T80I-HHF1::TRP1
H3T80S	MATa leu2Δ1 his3Δ200 ura3-52 trp1Δ63 lys2-1286 (hht1-hhf1)ΔLEU2 (hht2-hhf2)Δ::HIS3 Ty912Δ35-lacZ::his4 pHHT1T80S-HHF1::TRP1
H3T80V	MATa leu2Δ1 his3Δ200 ura3-52 trp1Δ63 lys2-1286 (hht1-hhf1)ΔLEU2 (hht2-hhf2)Δ::HIS3 Ty912Δ35-lacZ::his4 pHHT1T80V-HHF1::TRP1
H3T80Y	MATa leu2Δ1 his3Δ200 ura3-52 trp1Δ63 lys2-1286 (hht1-hhf1)ΔLEU2 (hht2-hhf2)Δ::HIS3 Ty912Δ35-lacZ::his4 pHHT1T80Y-HHF1::TRP1

Table S2. Primers used in this study.

Name	Sequence (from 5` to 3`)
H3K79R-F	GTCAGAGAAATCGCTCAAGATTTCAG
H3K79R-R	GATTGAAATCTCAAGTCGGTTCTGAA
H3K79N-F	GAGAAATCGCTCAAGATTTCAATACC
H3K79N-R	GATTGAAATCTCAAGTCGGTATTGAA
H3T80C-F	CGCTCAAGATTTCAAGTGCGACTTGAGATTTC
H3T80C-R	GAAATCTCAAGTCGCACTTGAAATCTTGAGCG
H3T80D-F	CGCTCAAGATTTCAAGAUCGACTTGAGATTTC
H3T80D-R	GAAATCGCTCAAGATTTCAAGATCGA
H3T80E-F	GACGAAGATTGAAATCTCAAGTCGAA
H3T80E-R	GAAATCGCTCAAGATTTCAAGTTCGA
H3T80F-F	CGCTCAAGATTTCAAGTTCGACTTGAGATTTC
H3T80F-R	GAAATCTCAAGTCGAACTTGAAATCTTGAGCG
H3T80I-F	GAGAAATCGCTCAAGATTTCAAGGAA
H3T80I-R	GATAGCAGAAGATTGAAATCTCAAGT
H3T80S-F	CGCTCAAGATTTCAAGTGCGACTTGAGATTTC
H3T80S-R	GAAATCTCAAGTCGCTCTTGAAATCTTGAGCG
H3T80V-F	CGCTCAAGATTTCAAGgtCGACTTGAGATTTC
H3T80V-R	GAAATCTCAAGTCGACCTTGAAATCTTGAGCG
H3T80Y-F	CGCTCAAGATTTCAAGTACGACTTGAGATTTC
H3T80Y-R	GAAATCTCAAGTCGTACTTGAAATCTTGAGCG
**Quantitative PCR**	
HML1-F	AGG TGT ATC GCA ATG GAA TG
HML1-R	AGT CCG TGC CGA AAA CTT TA
HML4-F	CGTCTAGCTGAGCATGTGAGG
HML4-R	ACTTCCAGACGCTATCCTGTGA
